# Cut-off points between pain intensities of the postoperative pain using receiver operating characteristic (ROC) curves

**DOI:** 10.1186/s12871-021-01245-5

**Published:** 2021-01-25

**Authors:** Sooyoung Cho, Youn Jin Kim, Minjin Lee, Jae Hee Woo, Hyun Jung Lee

**Affiliations:** 1 Department of Anesthesiology and Pain Medicine, Ewha Womans University College of Medicine, 260, Gonghang-daero, Gangseo-gu, Seoul, 07804 Republic of Korea; 2 Department of Anesthesiology and Pain Medicine, Ewha Womans University Medical Center Mokdong Hospital, Seoul, South Korea

**Keywords:** Analgesic drugs, Postoperative pain, ROC analysis, Surgery

## Abstract

**Background:**

Pain assessment and management are important in postoperative circumstances as overdosing of opioids can induce respiratory depression and critical consequences. We aimed this study to check the reliability of commonly used pain scales in a postoperative setting among Korean adults. We also intended to determine cut-off points of pain scores between mild and moderate pain and between moderate and severe pain by which can help to decide to use pain medication.

**Methods:**

A total of 180 adult patients undergoing elective non-cardiac surgery were included. Postoperative pain intensity was rated with a visual analog scale (VAS), numeric rating scale (NRS), faces pain scale revised (FPS-R), and verbal rating scale (VRS). The VRS rated pain according to four grades: none, mild, moderate, and severe. Pain assessments were performed twice: when the patients were alert enough to communicate after arrival at the postoperative care unit (PACU) and 30 min after arrival at the PACU. The levels of agreement among the scores were evaluated using intraclass correlation coefficients (ICCs). The cut-off points were determined by receiver operating characteristic curves.

**Results:**

The ICCs among the VAS, NRS, and FPS-R were consistently high (0.839–0.945). The pain categories were as follow: mild ≦ 5.3 / moderate 5.4 ~ 7.1 /severe ≧ 7.2 in VAS, mild ≦ 5 / moderate 6 ~ 7 / severe ≧ 8 in NRS, mild ≦ 4 / moderate 6 / severe 8 and 10 in FPS-R. The cut-off points for analgesics request were VAS ≧ 5.5, NRS ≧ 6, FPS-R ≧ 6, and VRS ≧ 2 (moderate or severe pain).

**Conclusions:**

During the immediate postoperative period, VAS, NRS, and FPS-R were well correlated. The boundary between mild and moderate pain was around five on 10-point scales, and it corresponded to the cut-off point of analgesic request. Healthcare providers should consider VRS and other patient-specific signs to avoid undertreatment of pain or overdosing of pain medication.

## Background

Up to 80% of patients experience acute postoperative pain, and the severity is greater than moderate in 75% of these patients [1]. Postoperative pain varies by type of surgery, comorbidities, age, sex, and patient expectations. Pain is an uncomfortable sensation or emotional experience related to actual or potential tissue damage [2, 3]. Inappropriate assessment or treatment of postoperative pain can cause anxiety, insomnia, emotional stress, and limited mobility. Moreover, it can result in negative outcomes such as deep vein thrombosis, atelectasis, pulmonary embolism, chronic pain, and delayed discharge or re-hospitalization. Pain can also lead to increased morbidity and mortality [4–6]. Therefore, postoperative pain should be controlled properly.

Pain assessment is necessary for the proper management of pain. There are several tools to assess the strength of pain validated intrapatient and inter-rater reliability. Visual analog scale (VAS), numeric rating scale (NRS), faces pain scale-revised (FPS-R), and face, legs, activity, cry, and consolability scale (FLACC) are scales that are commonly used in hospitals. The choice of tool is based on whether the patient can report his or her pain and varies according to developmental status, cognitive status, level of consciousness, education level, and cultural and language differences [1].

A specific value usually expresses these scales, and it cannot describe the tolerability of the pain to the individual patient. For example, when a patient responds to his or her pain as VAS 5.0 or NRS 5, we do not know if the patient perceives the pain as mild, moderate, or severe. There have been several studies that worked on categorizing the pain score or determining cut-off points focused on chronic pain such as cancer pain or musculoskeletal pain [7–11]. However, postoperative pain in a postoperative care unit (PACU) has different attributions when compared to other chronic pains in aspects of its acute onset after awakening from general anesthesia, interaction with remnant sedatives, and patient irritability. Delicate assessment of pain and management is important in the PACU as overdosing on opioids can induce respiratory depression and critical consequences. Therefore, we aimed this study to check the reliability of commonly used pain scales in a postoperative setting among Korean adults. Additionally, we assumed that patients would need analgesics when their pain was moderate or severe and intended to determine cut-off points of pain scores between mild and moderate pain and between moderate and severe pain.

## Methods

This prospective study was approved by the institutional review board of Ewha Womans University Medical Center, Seoul, Korea (EUMC 2016–01–019-001). We enrolled a total of 180 patients, each of whom provided written informed consent between April and October in 2018. Adult patients who were older than 19 years and were undergoing elective non-cardiac surgery were included. We excluded patients who had difficulty communicating, vision or hearing problems, those unable to move their arms after surgery, patients with previous histories of chronic pain and intake of pain medication, and those with cognitive disorders.

### Pain assessment

We collected four pain scores: VAS, NRS, FPS-R, and a verbal rating scale (VRS). For VAS, we offered a 10 cm line with a movable pointer so the patient could move the pointer to indicate his or her perceived pain severity on the line from no pain (0 cm) to the worst pain (10 cm). For NRS, the patients were asked to choose a fixed number, which represented their pain from 0 (no pain) to 10 (the worst pain). For FPS-R, we offered a sheet of paper on which there were six figures of faces of gradually changing facial expressions from a comforting face with no pain to a crying face indicating the worst pain. The patients were asked to choose one of the face figures that represented their pain best, and we recorded a corresponding score to the face: six even numbers from 0 for the comforting face to 10 for the crying face. For the VRS, we asked the patients to express their pain in the words of intensity: none, mild, moderate, severe (the score counted as 0, 1, 2, 3, respectively). The data were collected in the following order: VAS, NRS, FPS-R, and VRS.

Before arriving at an operating room, we educated the patients about how we would assess the four kinds of pain scores. An assigned anesthesiologist performed anesthesia, and the pain score assessments were performed by one investigator (M Lee) in the PACU. The pain score assessments were performed twice. The first pain assessment was when the patients opened their eyes spontaneously or responded well to physicians’ verbal commands to say their name after arriving at the PACU. The second pain assessment was performed 30 min after arriving at the PACU. During the stay at the PACU, analgesics were given on the patient’s request rather than the certain pain score, and every analgesia was given under the anesthesiologist’s confirmation to prevent respiratory depression or re-sedation. The choice of analgesic was the discretion of the assigned anesthesiologist, and the options were fentanyl 0.5 μg/kg or ketorolac tromethamine 30 mg.

### Sample size calculation

We hypothesized all the agreement levels between the three pain scores (VAS, NRS, and FPS-R) from the intraclass correlation coefficients (ICC) would be higher than 0.7. At the value of α = 0.05 and the power = 0.8, the sample size was calculated as 169 using the ICC Sample Size package for R. Considering potential attrition, we determined the target sample size to be 180.

### Statistical analysis

Agreement levels between the VAS and NRS, VAS and FPS-R, and NRS and FPS-R were analyzed by ICC applying a two-way random effects model using a consistency definition. Clinical significance of the ICC was as follows: less than 0.40 indicated poor agreement, between 0.40 and 0.59 was fair, between 0.60 and 0.74 was good, and more than 0.75 was considered excellent [12].

To determine the cut-off points of the scores, receiver operating characteristic (ROC) curve analyses were used. The ROC curve is a widely used method when figuring out a diagnostic test’s accuracy and a cut-off point of the test for a disease. This analysis draws a plot of sensitivity (true positive rate) by 1-specificity (false positive rate) at every test value by dichotomizing patients into having the disease or not. Then we can determine the test value where the sensitivity and specificity are highest as the cut-off point, which means that that value can classify whether the patient has disease best [13, 14].

Three ROC curves for each of the VAS, NRS, and FPS-R by VRS were drawn: for the borders of between none (VRS = 0) and mild pain (VRS = 1), between mild pain (VRS = 1) and moderate pain (VRS = 2), and between moderate pain (VRS = 2) and severe pain (VRS = 3). The distance to the corner determined the cut-off points. The distances (*d*) to the top-left corner from points on the ROC curve were calculated, and the point with the lowest distance was chosen for the cut-off point [14].

$$ d=\sqrt{{\left(1- sensitivity\right)}^2+{\left(1- sensitivity\right)}^2} $$

The result was considered significant when the *p*-value was smaller than 0.05. Statistical analyses were performed in SPSS version 26.0 for Windows (IBM, USA).

## Results

A total of one hundred and eighty patients completed the two sets of pain scores. One hundred and eighty-five patients were assessed for eligibility, and five patients declined to participate. There was no dropout of patients or missing data during the collecting of data. Demographic data and surgery-related data are shown in Table 1. All patients were Korean and used the Korean language. The mean age was 48.6 years, and 37.8% of the patients were male. Orthopedic surgery was the most common department (41.7%). Most of the patients (98.3%) received general anesthesia, and 7.8% of the patients received a nerve block procedure combined with general anesthesia. The mean operation time was 76.6 min, and the mean anesthesia time was 116.3 min. The patients stayed at PACU for about 40.0 min.

**Table 1:** Demographic and operation-related data

Age (years)	48.62 ± 13.4
Sex	
M	68 (37.8%)
F	112 (62.2%)
Height (cm)	164.2 ± 8.5
Weight (kg)	65.9 ± 13.4
BMI (kg/m^2^)	24.3 ± 3.7
ASA PS classification	
1	77 (42.8%)
2	100 (55.6%)
3	3 (1.7%)
Department	
General surgery	43 (23.9%)
Obstetrics and gynecology	28 (15.6%)
Orthopedic surgery	75 (41.7%)
Urology	15 (8.3%)
Other^a^	19 (10.5%)
Type of anesthesia	
General	177 (98.3%)
Regional	3 (1.7%)
Analgesic request	
Yes	64 (35.6%)
No	116 (64.4%)
PCA	
Yes	82 (45.6%)
No	98 (54.4%)
Duration of operation (minutes)	76.6 ± 56.7
Duration of anesthesia (minutes)	116.3 ± 59.8
Duration of PACU staying (minutes)	40.0 ± 13.1

Data are expressed by number (%) or mean ± SD. *ASA PS* American Society of Anesthesiologists Physical Status, *BMI* Body mass index, *PACU* Post-anesthesia care unit, *PCA* Patient-controlled analgesia

^a^Other departments include thoracic surgery, otorhinolaryngology, plastic surgery, oral and maxillofacial surgery, and neurosurgery

The distribution of the pain scores is presented in Table 2. More than 80% of the patients complained of mild or moderate pain (VRS = 1 or 2) at the two time points. The range of pain scores in mild pain (VRS = 1) was VAS 0 ~ 7.8, NRS 0 ~ 8, FPS-R 0 ~ 6. Among patients with moderate pain (VRS = 2), the ranges were VAS 1.8 ~ 9.4, NRS 3 ~ 9, FPS-R 2 ~ 10. For severe pain (VRS = 3), the ranges of pain scores were VAS 5.7 ~ 10, NRS 5 ~ 10, FPS-R 4 ~ 10. Among the patients who reported their pain intensity as none (VRS = 0), seven patients at the first pain assessment and five patients at the second pain assessment said at least one pain score was not zero. Vice versa, when the patients said they had mild pain (VRS = 1), one patient at the first pain assessment and two patients at the second pain assessment reported at least one pain score was zero.

**Table 2:** The range of pain scores in the post-anesthesia care unit (PACU)

	4PS	VAS	NRS	FPS-R
First pain assessment	None (*n* = 23)	0 (0–1)	0 (0–1)	0 (0–2)
	Mild (*n* = 87)	4.5 (0–7.8)	5 (0–8)	4 (2–6)
	Moderate (*n* = 59)	6.2 (1.8–9.3)	7 (3–9)	6 (2–10)
	Severe (*n* = 11)	8.6 (6–10)	9 (5–10)	8 (4–10)
Second pain assessment	None (*n* = 22)	0 (0–1.5)	0 (0–1)	0 (0–2)
	Mild (*n* = 77)	4.2 (1.0–7.5)	4 (1–7)	4 (0–6)
	Moderate (*n* = 71)	6.8 (3.1–9.4)	7 (4–9)	6 (2–8)
	Severe (*n* = 9)	10 (5.7–10)	10 (7–10)	10 (4–10)
Combined	None	0 (0–1.5)	0 (0–1)	0 (0–2)
	Mild	4.5 (0–7.8)	5 (0–8)	4 (0–6)
	Moderate	6.5 (1.8–9.4)	7 (3–9)	6 (2–10)
	Severe	9.15 (5.7–10)	9 (5–10)	8 (4–10)

Data are expressed by the median value (minimum - maximum). First pain assessment was performed when the patients opened their eyes spontaneously or responded well to physicians’ verbal commands to say their name after arriving at the PACU. The second pain assessment was performed 30 min after arriving at the PACU. *FPS-R* faces pain scale-revised, *NRS* Numerical rating scale, *PACU* Post-anesthesia care unit, *VAS* Visual analog scale, *4PS* Four-point scale

The levels of agreement among the VAS, NRS, and FPS-R are shown in Table 3. ICC between VAS and NRS was the highest among the three pairs of the scores (VAS and NRS, VAS and FPS-R, and NRS and FPS-R) at both time points, and the values were 0.917 and 0.945, respectively. All ICCs were over 0.8, indicating excellent agreement [12].

**Table 3:** Intraclass correlation coefficients (ICCs) among the pain scales

	VAS	NRS	FPS-R
First pain assessment			
VAS	–		
NRS	0.917 (0.891–0.938)	–	
FPS	0.858 (0.814–0.892)	0.839 (0.790–0.878)	–
Second pain assessment			
VAS	–		
NRS	0.945 (0.927–0.959)	–	
FPS	0.851 (0.805–0.887)	0.846 (0.799–0.883)	–

Data are expressed by ICC (95% CI). First pain assessment was performed when the patients opened their eyes spontaneously or responded well to physicians’ verbal commands to say their name after arriving at the PACU. The second pain assessment was performed 30 min after arriving at the PACU. *CI* Confident interval, *FPS-R* Faces pain scale-revised, *NRS* Numerical rating scale, *ICC* Intraclass correlation coefficient, *PACU* Post-anesthesia care unit, *VAS* Visual analog scale

**Fig. 1:**
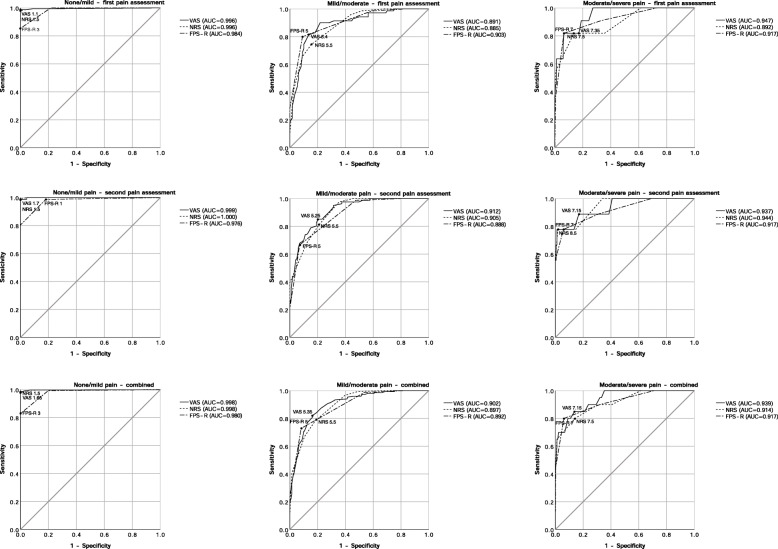
Receiver operating characteristic (ROC) curves of two time points. First pain assessment was performed when the patients opened their eyes spontaneously or responded well to physicians’ verbal commands to say their name after arriving at the PACU. The second pain assessment was performed 30 min after arriving at the PACU

The ROC curves to determine the cut-off points were drawn, and the cut-off points between none and mild pain, mild and moderate pain, and moderate and severe pain were determined (Fig. 1). and the categories were established (Table 4). At the first pain assessment, the cut-off points in order of none/mild pain, mild/moderate pain, and moderate/severe pain were 1.1, 5.4, and 7.35 in VAS, 1.5, 5.5, and 7.5 in NRS, and 3, 5, and 7 in FPS-R. When the second pain assessment, the cut-off points of these three boundaries were 1.7, 5.25, and 7.15 in VAS, 1.5, 5.5, and 8.5 in NRS, and 1, 5, and 7 in FPS-R. We repeated the same analysis with the 360 sets of data combining 180 sets of each time point, the cut-off points in order of none/mild pain, mild/moderate pain, and moderate/severe pain were 1.65, 5.35, and 7.15 in VAS, 1.5, 5.5, and 7.5 in NRS, and 3, 5, and 7 in FPS-R. All of these curves had the area under the curve (AUC) over 0.85, and all the *p*-values were less than 0.001.

**Table 4:** Categories of pain by the cut-off values

	VAS	NRS	FPS-R
First pain assessment			
None	≤ 1.1	≤ 1	0, 2
Mild	1.2–5.4	2–5	4
Moderate	5.5–7.3	6–8	6
Severe	7.4–10	9–10	8, 10
Second pain assessment			
None	≤ 1.7	≤ 1	0
Mild	≤ 5.2	2–5	2, 4
Moderate	5.3–7.1	6–8	6
Severe	7.2–10	9–10	8, 10
Combined			
None	≤ 1.6	≤ 1	0, 2
Mild	1.7–5.3	2–5	4
Moderate	5.4–7.1	6–7	6
Severe	7.2–10	8–10	8, 10

First pain assessment was performed when the patients opened their eyes spontaneously or responded well to physicians’ verbal commands to say their name after arriving at the PACU. The second pain assessment was performed 30 min after arriving at the PACU. *FPS-R* Faces pain scale-revised, *NRS* Numerical rating scale, *PACU* Post-anesthesia care unit, *VAS* Visual analog scale

**Fig. 2:**
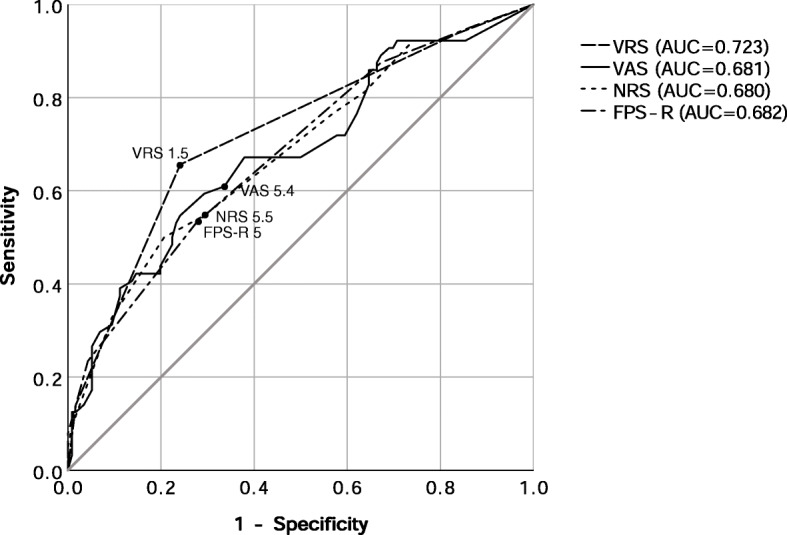
Receiver operating characteristic (ROC) curves for the cut-off point of the analgesic request

The ROC curve of the pain scores at the first pain assessment was drawn by the presence of analgesics injection during the stay in the PACU. The cut-off points in order of VAS, NRS, FPS-R, and VRS were 5.4, 5.5, 5, and 1.5. The AUCs were 0.681, 0.680, 0.682, and 0.723, respectively. All the *p*-values were less than 0.001 (Fig. 2). In other words, the patients requested an analgesic when their pain was VAS ≥ 5.5, NRS ≥ 6, FPS-R ≥ 6, or VRS ≥ 2 (moderate or severe pain).

## Discussion

The main results of our study were that the VAS, NRS, and FPS-R were well correlated in Korean adults. These results are similar to those of previous studies [15–20]. Second, we found the cut-off points of postoperative pain intensity using ROC curves. To our knowledge, this is the first study to determine cut-off points between degrees of pain intensity by ROC curve analysis. We observed the cut-off point between mild pain and moderate pain was around 5.5 on the VAS, which was higher than the values reported by most previous studies [7–11, 19, 21–24]. and physicians’ consensus.

In our study, patients with pain scores greater than around five required analgesics, and this value was similar to the boundary between mild and moderate pain. Cleeland et al. and Zelman et al. also suggested that the pain score NRS 5 was the cut-off point between “manageable and not manageable pain.” [10, 25] Although Dihle et al. and Zalon et al., who studied postoperative pain, suggested that an NRS greater than three should result in pain intervention, it also complied to moderate intensity of pain in their study [21, 23]. Our results also affirmed that pain greater than a mild intensity needs treatment. Delayed pain management after surgery could result in traumatic memory to a patient and even progressing to chronic pain or a mood disorder [26, 27]. Therefore, catching the timing of intervention for pain is important in the postoperative period.

There are several potential explanations for the higher cut-off point between mild and moderate pain. First, the time points were during the immediate postoperative period, and we asked patients to rate their current pain. On the contrary, previous studies evaluated the recalled pain (average pain, worst pain) minimums from anywhere from a few days to a maximum of a few years after the surgery [21, 23]. Second, the residual effects of anesthesia drugs and preoperative anxiety about post-surgical pain might increase the tolerance for pain. The third hypothetical reason is that we applied a different statistic method from previous studies. Previous studies used several sets consisted of possible cut-off points of mild/moderate pain and moderate/severe pain to determine the most optimal set of cut-off points with multivariate analyses of variance (MANOVA) [7, 8, 11]. However, this explanation is less likely because the median values of each intensity were also higher. The fourth reason is racial and cultural differences. Our study was conducted in the East Asian population while previous studies subjected to the Western population.

ROC analysis is an effective and one of the most commonly used methods to analyze the effectiveness of a diagnostic test and to find the optimal cut-off point [13, 14]. We used ROC curve analysis because we wanted to find the cut-off points without our guessing. The previous studies set pairs of cut-off points and chose one best-fit scheme among them [7, 8, 11]: for example, cut-off point (CP) Scheme 3,5 (with 1 to 3 classified as mild, 4 to 5 as moderate, and 6 to 10 as severe), CP Scheme 3,6, CP Scheme 4,6, CP Scheme 4,7, etc. This method could miss out on some pairs. Another disadvantage was that it rounds off the decimal of VAS, which our methods did not have to take. Moreover, by using the ROC analysis, we could find the cut-off point between no pain and mild pain. However, when the number of patients was small in no pain and severe pain, the ROC curves between none/mild pain and moderate/severe pain were not round and had inflections, as seen in Fig. 1. After combining the data of two time points, we could get more rounded the ROC curve.

Racial and ethnic differences in pain or treatment have been consistently reported [28–32]. Several studies found that Asians had a lower tolerance to pain with higher pain scores than Caucasians [28–30]. Most of the studies drew their study cohorts from multiracial countries. In these countries, Asians are a minority population; therefore, these studies might have biased results because of a language barrier or communicational difficulties. This study subjected only Korean people who speak Korean as their native language. Our results suggest that Asians might report higher pain scores in the same category as other races. It is an important point because this fact might affect medication. Kennel et al. pointed out that the language barrier might limit pain evaluation in minority patients and could lead to less effective treatment for pain [32]. We hope our results help Western physicians understand Asian patients’ pain behaviors.

We observed a wide range of pain scores within one pain intensity. For example, mild pain ratings ranged from 0 to 8 on the NRS and 0 to 7.8 on the VAS. It shows that pain assessments could be superficial and incomplete when they are performed using only pain scores. Healthcare providers are prone to write pain scores less than 3 or 4 as mild pain. However, some patients may perceive their pain to be moderately intense, and these patients may need a pain-reducing drug. Vice versa, even when pain scores are as high as 7 or 8, some patients might think their pain is mild and tolerable. Therefore, doctors or nurses should approach pain in multiple ways to determine whether their patients’ pain is tolerable or needs intervention. Considering we found that the cut-off point between mild and moderate pain was around 5.5, we have to be cautious when we give opioids to the patients with NRS ratings of 4 or 5. The immediate postoperative period is unique due to the residual effects of anesthesia drugs. Providers should be attentive following drug administration. Therefore, from the fact that the AUC of the ROC curve for the analgesic request was the biggest in VRS (0.723), pain assessment using VRS would be useful before an administration of analgesics.

This study has several limitations. First, we did not study the patients’ preferences. Second, we included a heterogeneous group of patients undergoing various surgeries. Prediction of postoperative pain intensity is affected by various factors such as age, surgery type, preexisting pain, and anxiety [33–35]. However, our results are generally applicable values because they did not represent a specific type of surgery or a specific group of patients. Third, the pain score at the first pain assessment might not be the same as the pain score when patients request medication, and it was analyzed regardless of patient-controlled analgesia. The low AUC of ROC curves of pain medication might be because the timing of the pain assessments did not exactly correspond to when the patients claimed they needed medication. Forth, it might be helpful if we compared the cut-off values between the immediate postoperative period and the postoperative period days after surgery to evaluate the residual effect of anesthetics.

## Conclusion

During the immediate postoperative period, any pain scale among the VAS, NRS, or FPS-R can be used. We recommend combining these scales with the VRS and other patient-specific signs to avoid under- or overdosing of pain medication, particularly for opioids. We suggest a simple, applicable category of pain in the immediate postoperative situation: an NRS score of 5 or lower is mild pain, an NRS score of 6 to 7 is moderate pain, and an NRS score of 8 to 10 indicates severe pain.

## Data Availability

The datasets analyzed during the current study are available from the corresponding author upon reasonable request.

## Abbreviations

AUC: Area under the curve
FLACC: Face, legs, activity, cry, and consolability scale
FPS-R: Faces pain scale revised
ICC: Intraclass correlation coefficients
MANOVA: Multivariate analyses of variance
PACU: Postoperative care unit
ROC: Receiver operating characteristic
VAS: Visual analog scale
VRS: Verbal rating scale
