# Correction to: Cut-off points between pain intensities of the postoperative pain using receiver operating characteristic (ROC) curves

**DOI:** 10.1186/s12871-021-01410-w

**Published:** 2021-07-16

**Authors:** Sooyoung Cho, Youn Jin Kim, Minjin Lee, Jae Hee Woo, Hyun Jung Lee

**Affiliations:** 1 Department of Anesthesiology and Pain Medicine, Ewha Womans University College of Medicine, 260, Gonghang-daero, Gangseo-gu, Seoul, 07804 Republic of Korea; 2 Department of Anesthesiology and Pain Medicine, Ewha Womans University Medical Center Mokdong Hospital, Seoul, South Korea

**Correction to: BMC Anesthesiol 21, 29 (2021)**

**https://doi.org/10.1186/s12871-021-01245-5**

Following publication of the original article [1], the authors report an error in the Method section. The formula used for determining the cut-off points from the receiver operating characteristic (ROC) curves was published incorrectly, so the authors correct the error. The formula was published as below originally.

$$d=\sqrt{{\left(1-sensitivity\right)}^{2}+{\left(1-sensitivity\right)}^{2}}$$

The authors correct this as below [2].

$$d=\sqrt{{\left(1-sensitivity\right)}^{2}+{\left(1-specificity\right)}^{2}}$$
